# Association between urinary levels of 8-hydroxy-2-deoxyguanosine and F_2a_-isoprostane in male football players and healthy non-athlete controls with dietary inflammatory and antioxidant indices

**DOI:** 10.3389/fnut.2022.1101532

**Published:** 2023-01-24

**Authors:** Mahsa Zare, Zainab Shateri, Mehran Nouri, Parvin Sarbakhsh, Mohammad Hasan Eftekhari, Bahram Pourghassem Gargari

**Affiliations:** ^1^Student Research Committee, Faculty of Nutrition and Food Science, Tabriz University of Medical Sciences, Tabriz, Iran; ^2^Student Research Committee, Ahvaz Jundishapour University of Medical Science, Ahvaz, Iran; ^3^Department of Community Nutrition, School of Nutrition and Food Science, Shiraz University of Medical Sciences, Shiraz, Iran; ^4^Students' Research Committee, Shiraz University of Medical Sciences, Shiraz, Iran; ^5^Health Policy Research Center, Institute of Health, Shiraz University of Medical Sciences, Shiraz, Iran; ^6^Department of Statistics and Epidemiology, School of Public Health, Tabriz University of Medical Sciences, Tabriz, Iran; ^7^Department of Clinical Nutrition, School of Nutrition and Food Sciences, Shiraz University of Medical Sciences, Shiraz, Iran; ^8^Nutrition Research Center, Department of Biochemistry and Diet Therapy, Faculty of Nutrition and Food Sciences, Tabriz University of Medical Sciences, Tabriz, Iran

**Keywords:** oxidative stress, 8-hydroxy-2-deoxyguanosine, F_2alpha_-isoprostane, dietary inflammatory index, dietary antioxidant capacity, dietary phytochemical index, football

## Abstract

**Purpose:**

The relationship between the inflammatory and antioxidant potential of an athlete's diet and their oxidative biomarkers is an important area of investigation. Therefore, this study aimed to assess the excretion of 8-hydroxy-2-deoxyguanosine (8-OHdG) and F_2alpha_-isoprostane (F_2a_-IP) in the urine of male football players and healthy non-athlete controls. This study also aimed to examine the associations among the dietary inflammatory index (DII), the dietary total antioxidant capacity (DTAC), and the dietary phytochemical index (PI) with 8-OHdG and F_2a_-IP.

**Methods:**

In this descriptive-analytical study, 45 male football players and 45 healthy non-athletes, who were individually matched based on age and body mass index (BMI), were recruited from Shiraz City, Iran. Fasted urine samples were analyzed for 8-OHdG and F_2a_-IP levels. Anthropometric measurements were performed, and body composition was assessed using a body composition analyzer. A valid food frequency questionnaire (FFQ) was used to calculate DII, DTAC, and PI scores. Data analysis was conducted using a generalized estimating equation (GEE) model.

**Results:**

We found that 8-OHdG (β = −6.96), F_2a_-IP (β = −82.58), and DII (β = −2.06) were significantly lower, while DTAC (β = 2.37) and PI (β = 0.084) were significantly higher in the football player group compared with the non-athlete group (*P* < 0.001 for all variables). In all participants, dietary indices were significantly associated with oxidative biomarkers. DII was positively associated with 8-OHdG (β = 2.25; *P* < 0.001) and F_2a_-IP (β = 38.34; *P* < 0.001). Furthermore, negative associations between DTAC (β = −1.42; *P* < 0.001) and PI (β = −35.37; *P* < 0.001) with 8-OHdG were found. Moreover, DTAC (β = −17.34; *P* < 0.001) and PI (β = −428.11; *P* = 0.003) were negatively associated with F_2a_-IP.

**Conclusion:**

The results of this study highlighted the importance of a healthy diet in reducing oxidative stress among football athletes. The levels of urinary biomarkers for DNA and lipid oxidation were found to be lower in football players compared to non-athletes. This suggests that following an anti-inflammatory and antioxidant-rich diet may help reduce oxidative stress in these individuals.

## Introduction

Football, the world's most popular sport (1), is a team endurance-speed sport that involves jumps, sprints, changes of direction, and high-intensity running; it relies on both aerobic and anaerobic energy pathways. Consequently, these demanding physical efforts lead to the development of inflammation, muscle damage, and oxidative stress (OS) (2–5).

OS is defined as an unfavorable imbalance between the production of reactive oxygen species (ROS) and antioxidant defense that causes damage to cellular DNA, lipids, and proteins, which can result in reduced athletic performance and impaired recovery (6, 7). The relationship between exercise-induced ROS and cellular damage has been explained by the hormesis theory, which is a bell-shaped curve in which regular/moderate exercise leads to positive consequences. In contrast, high/strenuous exercise leads to negative consequences (8). Oxidative stress-derived DNA and lipid damages can be measured by two reliable biomarkers: urinary excretion rates of 8-hydroxy-2-deoxyguanosine (8-OHdG) (6) and F_2alpha_-isoprostane (F_2a_-IP) (9), respectively.

8-OHdG formed from hydroxyl radical attack at the C-8 position of deoxyguanosine (6) and F_2a_-IP generated from free radical-catalyzed peroxidation of arachidonic acid (9). Several studies have suggested an association between increased levels of urinary 8-OHdG and F_2a_-IP with some diseases such as cancer, diabetes, and atherosclerosis (10). Factors that have been associated with the levels of urinary 8-OHdG and F_2a_-IP are body mass index (BMI), age, gender, smoking, dietary factors, and exercise (6, 10). A meta-analysis study concluded that a significant increase in 8-OHdG levels remains between 2 h and one day after acute aerobic exercise but not within 5–28 days post-exercise (11). Another review study summarized that “acute exercise leads to a short-lived increase in plasma and skeletal muscle F_2a_-IP levels, but regular exercise reveals a trend for decreased urinary F_2a_-IP levels” (12).

Given the body of evidence that supports the relationship between inflammation status and 8-OHdG and F_2a_-IP concentrations (13), as well as the evidence for the role of various dietary factors and different food groups in the regulation of inflammatory processes (14, 15), it seems that assessment of the dietary inflammatory index (DII) as a validated tool for predicting the inflammatory potential of diet (16) is useful in expanding our knowledge in this field.

In addition, some previous studies demonstrated that urinary excretion rates of 8-OHdG and F_2a_-IP have been linked to dietary antioxidant nutrients and antioxidant-rich foods (10, 17). In order to assess the overall dietary and plasma status of antioxidants, dietary total antioxidant capacity (DTAC) is a useful, validated tool (18). In this line, a study has reported a negative correlation between DTAC and the levels of 8-isoprostaglandin F_2alpha_ in athletes (19). Moreover, the dietary phytochemical index (PI) is a suitable tool to evaluate the received amount of phytochemicals (20). Phytochemicals, which are bioactive chemicals in antioxidant-rich foods, as anti-inflammatory agents and antioxidants, play beneficial roles in OS, immunity, and inflammation status (21).

To our knowledge, to date, no single study has been conducted regarding the association between urinary biomarkers of oxidative stress and dietary pro-inflammatory and antioxidant indices in football players. Given the limited data, the objective of the present study was to assess the urinary excretions of 8-OHdG and F_2a_-IP and also evaluate DII, DTAC, and PI scores, as well as investigate the relationships between the abovementioned dietary indices with 8-OHdG and F_2a_-IP in male football players and their healthy non-athlete controls.

## Materials and methods

### Study design and participants

The present study was a descriptive-analytical investigation that compared two groups of 90 men (45 football players and 45 healthy non-athlete controls). The two groups were matched based on age and BMI. The football players and non-athletes were recruited through cluster sampling among 36 football clubs in Shiraz City, Iran, and 10 schools at Shiraz University of Medical Sciences, respectively. Among the football clubs, five clubs were randomly selected, and nine football players who met the inclusion criteria were randomly included from each selected club. Likewise, five schools were randomly selected among Shiraz University's schools, and after that, nine eligible persons were randomly included from each selected school.

The inclusion criteria for football players consist of (1) the willingness of subjects to participate in this research and complete the consent form; (2) individuals aged 20–30 years with a BMI of 20–25 (kg/m^2^); (3) football experience at least for the last 2–3 years and following the training protocol of 3–4 days/week and 90–120 min/session; (4) a metabolic equivalent of task (MET) of more than 3,000 (min/week); (5) stability in eating behaviors and weight within the last 2 months; (6) not taking omega-3 and antioxidant supplements within the last month; (7) no alcohol or smoking; and (8) giving up caffeine within 24 h before urine sampling. The inclusion criteria for non-athletes included the willingness of subjects to participate and sign the consent form, individuals matching with football players (based on age and BMI), a MET between 600 and 3,000 (min/week), and items #5-8, the criteria mentioned above for the football player group.

The following were regarded as exclusion criteria for both groups: (1) the presence of infectious, inflammatory, cardiovascular, liver, kidney, and respiratory diseases, hypertension, high blood lipids, stroke, thyroid problems, and malignancies; (2) taking drugs that alter the metabolism of oxidants and antioxidants in the past month; (3) being a consumer of non-steroidal anti-inflammatory drugs (NSAIDs); (4) diseases that affect the oxidation of nucleic acids, such as diabetes, hemochromatosis, schizophrenia, and bipolar disorder; (5) hormone therapy; (6) smoking and drinking alcohol; and (7) those who answered < 90% of items on the food frequency questionnaire (FFQ).

### Sample size

The sample size was calculated using G-power software based on the mean difference in the urinary level of 8-OHdG between athlete and non-athlete groups from Rahimi et al.'s study (22). Due to the existing correlation between matched athletes and non-athletes, the sample size was calculated based on paired-design studies by considering a 0.15 correlation between the urinary level of 8-OHdG in matched athletes and non-athletes, 80% power, and 95% confidence. The required sample size for each group was 43 participants. By considering about 5% of the sample drop, the final sample size for each group increased to 45 participants.

### Measurements

The inclusion criteria were assessed on the first visit, and then anthropometric measurements, a dietary assessment, and urine samples were taken from eligible participants on the second visit. Interviewer-administered questionnaires were used to collect personal anthropometric, physical activity, medical history, general nutrition, and dietary intake information. To prevent measurement errors, all items were measured by the same person. Anthropometric measures and urine sampling were obtained in the morning.

The height and weight were measured using a wall-mounted Seca stadiometer and a digital Seca scale (with a sensitivity of 0.1 cm and 0.1 kg) (Seca, Germany), respectively, while the participant wore light clothes without shoes. BMI was considered to be weight (kg) divided by height squared (m^2^). Waist circumference (WC) was measured using an inelastic tape at the point midway between the lowest rib and the iliac crest at the end of a normal exhalation without imposing any pressure on the body surface to the nearest 0.1 cm. Hip circumference (HC) was measured at the maximum level of buttock extension using an inelastic tape measure (with a precision of 0.1 cm). Waist-to-hip ratio (WHR) was calculated by dividing WC (cm) by HC (cm).

Body composition, including fat mass (FM), fat-free mass (FFM), total body water (TBW), and total body fat (TBF), was estimated using the InBody 270 body composition analyzer. Participants were instructed to follow certain guidelines before the test: (1) avoiding heavy physical activity during the last 3 days; (2) adequate hydration during the last 2 h; (3) abstaining from caffeine and large meals during the last 24 and 12 h, respectively; (4) emptying their bladder. Physical activity was calculated using the validated short form of the international physical activity questionnaire (IPAQ) (23) and expressed as the MET hours per week (MET-h/week).

### Laboratory assays

After a 12-h fasting period, morning urine samples were collected from 8–10 (a.m.) from all participants (for football players, 3 days after the last football training). Urinary levels of 8-OHdG and F_2a_-IP were determined by an enzyme-linked immunosorbent assay (ELISA) using commercial kits (the 8-OHdG kit, abx150312, Abbexa, United Kingdom, and the 8-epi PGF_2a_ kit, abx150311, Abbexa, United Kingdom) according to the manufacturer's instructions. Values for 8-OHdG and F_2a_-IP were normalized by creatinine measured in urine (spectrophotometry creatinine test kit, Pars Azmoon, Tehran, Iran).

### Assessment of dietary intake

Dietary intake over the previous year was obtained by applying a valid 168-item, semi-quantitative FFQ (24) *via* a face-to-face interview with a trained dietitian. This questionnaire reported the frequency of consumption of each food item on a daily, weekly, monthly, or yearly basis. Next, the reported frequency of each food intake was converted to a gram per day using household measures (25). Then, Nutritionist IV software (First Databank Division, The Hearst Corporation, San Bruno, CA, USA) modified for Iranian foods was used to calculate the energy and nutrient content of each food item.

The DII, DTAC, and PI were calculated according to the dietary data derived from the FFQ.

### Calculation of DII

The DII score was calculated based on the method proposed in the study by Shivappa et al. (16). In the current study, we used 31 food parameters to calculate DII [energy, carbohydrate, protein, fat, vitamin B_12_, vitamin B_9_, vitamin B_6_, vitamin B_3_, vitamin B_2_, vitamin B_1_, beta-carotene, vitamin A, vitamin C, vitamin D, vitamin E, fiber, cholesterol, saturated fat, trans fat, monounsaturated fatty acids (MUFA), polyunsaturated fatty acids (PUFA), omega_3_, omega_6_, iron, zinc, selenium, magnesium, caffeine, garlic, onion, and tea].

Initially, energy-adjusted amounts of each nutrient were estimated using the residual method (26). Then, the z-score was calculated by subtracting the “standard global mean” from the actual food parameter value and dividing it by its “global standard deviation.” After that, the z-score was converted to a percentile and centered by multiplying by two and subtracting one score in order to minimize the effect of outliers or right-skewing, as earlier studies did. This value was then multiplied by the respective food inflammatory effect score to obtain the food parameter-specific DII score. Then, all of them were summed up to create the overall DII score for each participant. A more positive DII score indicates a greater pro-inflammatory potential of the participant's diet.

### Calculation of DTAC

DTAC was calculated based on the ferric-reducing antioxidant power (FRAP) values from the study by Carlsen et al. (27). The FRAP assay measures the potential of dietary antioxidants to reduce Fe^3+^ to Fe^2+^ ions and is expressed as mmol per 100 g of food (mmol/100 g) (28). At first, the frequency of consumption of each food item (gr) was multiplied by its related antioxidant capacity value per 100 g and then summed to obtain the total dietary antioxidant capacity. For similar food items, for example, several types of bread, the overall mean value of DTAC was considered. For food items for which data were not available, the value of the nearest comparable food was assigned.

### Calculation of PI

As developed by McCarty (20), the PI was calculated as the percentage of the daily dietary energy derived from phytochemical-rich foods (kcal) divided by the total daily energy intake (kcal) [PI = (phytochemical kcal/total kcal) ^*^ 100]. Phytochemical-rich foods considered in this study were as follows: whole grains, nuts, legumes, seeds, vegetables, fruits, and others (olive oil, tea, and coffee).

### Statistical analysis

Statistical Package for Social Sciences (SPSS) version 25.0 was applied to perform statistical data analysis. The normality of the data distribution was evaluated by the Kolmogorov and Smirnov tests. The characteristics of all participants are expressed as mean ± standard deviation (SD) for normally distributed variables. By considering matching design in data analysis, a generalized estimating equation (GEE) model with an identity link function and an exchangeable correlation structure was performed for all data analysis. For comparing the mean values of baseline characteristics, DII items, and PI components in study groups, a suited GEE model was employed. In order to assess the differences of 8-OHdG, F_2a_-IP, DII, DTAC, and PI in the football player group in comparison to the non-athlete group, a suited GEE approach that adjusted for potential confounders (FM, FFM, and WHR) was undertaken. The association of DII, DTAC, and PI with 8-OHdG and F_2a_-IP in all participants was analyzed by linear regression analysis with the GEE. *P* < 0.05 were considered statistically significant.

## Results

The participant's characteristics are summarized in Table 1. The mean age of participants was 22.88 years (SD = 2.41), and their BMI was 22.08 kg/m^2^ (SD = 1.35). The mean values of FFM (kg), TBW (L), MET (h/week), DTAC (mmol/100 g), and PI (%Energy) were significantly higher in the football player group, while FM (kg), TBF (%), WHR, DII, and urinary levels of 8-OHdG (ng/mg creatinine) and F_2a_-IP (pg/mg creatinine) were significantly lower in the football player group compared with the non-athlete group (*P* < 0.001 for all values).

**Table 1:** Baseline characteristics of the study participants.

**Variable**	**Football players**	**Non-athletes**	**All participants**	***P***
	**(*n* = 45)**	**(*n* = 45)**	**(*n* = 90)**	
	**(mean ±SD)**	**(mean ±SD)**	**(mean ±SD)**	
Age (years)	22.89 ± 2.42	22.87 ± 2.42	22.88 ± 2.41	–
BMI (kg/m^2^)	22.06 ± 1.34	22.09 ± 1.37	22.08 ± 1.35	–
Height (cm)	175.18 ± 6.63	173.22 ± 10.63	174.2 ± 8.86	0.29
Weight (kg)	67.85 ± 7.03	66.80 ± 10.41	67.33 ± 8.85	0.47
FM (kg)	9.60 ± 3.12	13.10 ± 4.74	11.35 ± 4.36	**< 0.001**
FFM (kg)	58.25 ± 6.05	53.76 ± 6.98	56 ± 6.88	**< 0.001**
TBF (%)	14.07 ± 3.85	19.15 ± 4.69	16.61 ± 4.97	**< 0.001**
TBW (L)	42.59 ± 4.45	37.91 ± 5.36	40.25 ± 5.44	**< 0.001**
WHR	0.79 ± 0.02	0.86 ± 0.05	0.83 ± 0.05	**< 0.001**
MET (h/week)	62.68 ± 9.04	16.21 ± 6.26	39.44 ± 24.61	**< 0.001**
8-OHdG (ng/mg creatinine)	10.90 ± 3.66	20.30 ± 9.52	15.6 ± 8.59	**< 0.001**
F_2a_-IP (pg/mg creatinine)	124.51 ± 41.46	241.6 ± 112.38	183.06 ± 102.76	**< 0.001**
DII	−0.99 ± 1.15	0.86 ± 1.44	−0.06 ± 1.59	**< 0.001**
DTAC (mmol/100 g)	7.79 ± 2.3	5.31 ± 1.36	6.53 ± 2.25	**< 0.001**
PI (%Energy)	30.69 ± 7.45	22.96 ± 7.80	26.83 ± 8.52	**< 0.001**

BMI, body mass index; FM, fat mass; FFM, fat-free mass; TBF, total body fat; TBW, total body water; WHR, waist-hip ratio; MET, metabolic equivalent; 8-OHdG, 8-hydroxy-2-deoxy guanosine; F_2a_-IP, F_2a_-isoprostane; DII, dietary inflammatory index; DTAC, dietary total antioxidant capacity; PI, phytochemical index.

Using the GEE model with identity link function and exchangeable correlation structure (study groups were matched for age and BMI).

Bold p-value (< 0.05) is considered as statistically significant.

The DII and PI components of the subject groups are shown in Table 2. Significant differences were detected among some components. Among the average daily intakes of various DII components; energy (Kcal), carbohydrate (g), protein (g), vitamin B_9_ (mg), vitamin B_6_ (mg), vitamin B_3_ (mg), vitamin B_2_ (mg), vitamin B_1_ (mg), beta-carotene (mg), vitamin A (mg), vitamin C (mg), vitamin D (ug), fiber (g), cholesterol (mg), iron (mg), Mg (mg), Se (mg), Zn (mg), caffeine (mg), garlic (g), onion (g), and also among PI components; fruits (%Energy) and others (olive oil, tea, and coffee) (%Energy) were higher in the football player group [*P* < 0.001 for all values except vitamin A (*P* = 0.003), vitamin C (*P* = 0.002), fiber (*P* = 0.003), onion (*P* = 0.004), fruits (*P* = 0.009)], while vitamin E (mg) (*P* = 0.002), MUFA (g) (*P* = 0.01), trans fat (g) (*P* = 0.001), and omega_6_ (mg) (*P* = 0.03) were higher in the non-athlete group.

**Table 2:** Nutrients and food items of the study participants.

**Variable**	**Football players (*n* = 45) (mean ±SD)**	**Non-athletes (*n* = 45) (mean ±SD)**	**All participants (*n* = 90) (mean ±SD)**	***P***
**DII components**				
Energy (Kcal)	2,563.12 ± 136.99	2,354.31 ±130.38	2,458.72 ± 169.42	**< 0.001**
Carbohydrate (g)	365.67 ± 31.12	314.67 ± 22.10	340.17 ± 37.12	**< 0.001**
Protein (g)	82.54 ± 7.68	70.60 ± 8.60	76.57 ± 10.09	**< 0.001**
Fat (g)	90.88 ± 9.84	94.06 ± 9.50	92.47 ± 9.75	0.07
Vitamin B_12_ (mg)	5.22 ± 1.77	4.94 ± 1.93	5.08 ± 1.85	0.49
Vitamin B_9_ (mg)	668.37 ± 53.04	577.0 ± 59.50	622.69 ± 72.47	**< 0.001**
Vitamin B_6_ (mg)	1.70 ± 0.17	1.47 ± 0.20	1.59 ± 0.22	**< 0.001**
Vitamin B_3_ (mg)	23.85 ± 2.03	21.40 ± 2.40	22.62 ± 2.53	**< 0.001**
Vitamin B_2_ (mg)	2.11 ± 0.29	1.68 ± 0.23	1.89 ± 0.34	**< 0.001**
Vitamin B_1_ (mg)	2.26 ± 0.23	1.98 ± 0.19	2.12 ± 0.25	**< 0.001**
Beta-carotene (mg)	2,998.59 ± 1113.33	2,149.84 ± 804.54	2,574.21 ± 1055.89	**< 0.001**
Vitamin A (mg)	682.31 ± 215.94	541.20 ± 203.72	611.75 ± 220.46	**0.003**
Vitamin C (mg)	112.61 ± 36.13	91.35 ± 33.81	101.98 ± 36.40	**0.002**
Vitamin D (ug)	2.47 ± 0.99	1.25 ± 0.62	1.86 ± 1.03	**< 0.001**
Vitamin E (mg)	21.90 ± 5.39	25.43 ± 5.11	23.66 ± 5.51	**0.002**
Fiber (g)	56.20 ± 10.08	49.42 ± 10.54	52.81 ± 10.81	**0.003**
Cholesterol (mg)	406.23 ± 152.16	292.15 ± 75.30	349.19 ± 132.44	**< 0.001**
MUFA (g)	31.30 ± 4.01	33.38 ± 4.22	32.34 ± 4.23	**0.01**
PUFA (g)	22.70 ± 3.95	24.03 ± 3.07	23.36 ± 3.58	0.06
SFA (g)	24.05 ± 3.26	24.75 ± 3.31	24.40 ± 3.28	0.31
Trans (g)	0.1 ± 0.03	0.3 ± 0.07	0.2 ± 0.04	**0.001**
Omega_3_ (mg)	1.01 ± 0.32	1.02 ± 0.26	1.02 ± 0.29	0.88
Omega_6_ (mg)	20.63 ± 3.82	22.10 ± 2.97	21.36 ± 3.48	**0.03**
Iron (mg)	19.79 ± 1.69	17.23 ± 1.56	18.51 ± 2.07	**< 0.001**
Mg (mg)	399.25 ± 55.87	315.27 ± 51.35	357.26 ± 68.04	**< 0.001**
Se (mg)	131.18 ± 17.84	104.06 ± 16.57	117.62 ± 21.88	**< 0.001**
Zn (mg)	12.27 ± 1.91	10.47 ± 1.58	11.37 ± 1.96	**< 0.001**
Caffeine (mg)	62.40 ± 38.14	35.19 ± 23.16	48.80 ± 34.23	**< 0.001**
Garlic (g)	1.36 ± 1.21	0.39 ± 0.27	0.87 ± 0.105	**< 0.001**
Onion (g)	11.42 ± 11.24	5.92 ± 4.49	8.67 ± 0.94	**0.004**
Tea (g)	182.08 ± 161.18	125.82 ± 109.45	153.95 ± 139.88	0.07
**PI components (%Energy)**				
Whole grains	5.18 ± 4.47	4.07 ± 4.96	4.62 ± 0.49	0.26
Nuts	1.64 ± 0.25	1.39 ± 0.23	1.51 ± 0.17	0.48
Legumes	4.26 ± 0.86	4.33 ± 1.03	4.30 ± 0.94	0.73
Seeds	3.27 ± 0.52	2.28 ± 0.21	2.77 ± 2.74	0.09
Vegetables	3.89 ± 1.23	3.68 ± 1.16	3.78 ± 1.19	0.43
Fruits	7.54 ± 3.36	5.93 ± 2.74	6.74 ± 3.15	**0.009**
Others	4.89 ± 4.74	1.26 ± 0.20	3.07 ± 0.41	**< 0.001**

DII, dietary inflammatory index; Mg, magnesium; MUFA, monounsaturated fatty acid; PUFA, polyunsaturated fatty acid; SFA, saturated fatty acid; Se, selenium; Zn, zinc.

Others are olive oil, tea, and coffee.

Using the GEE model with identity link function and exchangeable correlation structure (study groups were matched for age and BMI).

Bold p-value (< 0.05) is considered as statistically significant.

The comparison of urinary parameters and questionnaire indices revealed differences between the two groups after considering the adjustment for confounders (FM, FFM, and WHR) are shown in Table 3. These results indicated that the football player group was significantly lower in 8-OHdG (ng/mg creatinine) (β = −6.96), F_2a_-IP (pg/mg creatinine) (β = −82.58), and DII (β = −2.06), and significantly higher in DTAC (mmol/100 g) (β = 2.37) and PI (%Energy) (β = 0.084) than the non-athlete group (*P* < 0.001 for all variables).

**Table 3:** GEE results for comparing oxidative biomarkers and dietary indices between football player and non-athlete groups.

**Variables**		**B**	**SE**	**95% Wald confidence interval** **(lower, upper)**	***P***
8-OHdG (ng/mg creatinine)	Non-athletes	Reference	–	–	–
	Football players	−6.96	1.76	(−10.43, −3.49)	**< 0.001**
F_2a_-IP (pg/mg creatinine)	Non-athletes	Reference	–	–	–
	Football players	−82.58	19.79	(−121.38, −43.79)	**< 0.001**
DII	Non-athletes	Reference	–	–	–
	Football players	−2.06	0.35	(−2.75, −1.37)	**< 0.001**
DTAC (mmol/100 g)	Non-athletes	Reference	–	–	–
	Football players	2.37	0.49	(1.4, 3.35)	**< 0.001**
PI (%Energy)	Non-athletes	Reference	–	–	–
	Football players	0.084	0.019	(0.046, 0.12)	**< 0.001**

8-OHdG, 8-hydroxy-2-deoxy guanosine; F_2a_-IP, F_2a_-isoprostane; DII, dietary inflammatory index; DTAC, dietary total antioxidant capacity; PI, phytochemical index.

Using the GEE model with identity link function and exchangeable correlation structure and adjusted for FM, FFM, and WHR (study groups were matched for age and BMI).

Bold p-value (< 0.05) is considered as statistically significant.

The results of the linear regression analysis for the associations among DII, DTAC, and PI with 8-OHdG in all participants are presented in Table 4. Significant associations were detected. The results revealed that DII was positively associated with 8-OhdG; as DII increased by one unit, 8-OHdG (ng/mg creatinine) increased by 2.25 (*P* < 0.001). Moreover, negative associations were shown between DTAC and PI with 8-OHdG; as DTAC (mmol/100 g) and PI (%Energy) increased by one unit, 8-OHdG (ng/mg creatinine) decreased by 1.42 (*P* < 0.001) and 35.37 (*P* < 0.001), respectively.

**Table 4:** Linear regression model using the GEE method for assessing the association of DII, DTAC, and PI with 8-OHdG in all participants.

**Variables**	**B**	**SE**	**95% Wald confidence interval** **(lower, upper)**	***P***
DII	2.25	0.55	(1.16, 3.39)	**< 0.001**
DTAC (mmol/100 g)	−1.42	0.28	(−1.98, −0.85)	**< 0.001**
PI (%Energy)	−35.37	9.72	(−54.44, −16.3)	**< 0.001**

DII, dietary inflammatory index; DTAC, dietary total antioxidant capacity; PI, phytochemical index.

Using the GEE model with identity link function and exchangeable correlation structure (study groups were matched for age and BMI).

Bold p-value (< 0.05) is considered as statistically significant.

Table 5 shows the results of the linear regression analysis for the association of DII, DTAC, and PI with F_2a_-IP in all participants. Significant associations were found. Results indicate that DII was positively associated with F_2a_-IP; as DII increased by one unit, F_2a_-IP (pg/mg creatinine) increased by 28.34 (*P* < 0.001). In addition, negative associations were observed between DTAC and PI with F_2a_-IP; as DTAC (mmol/100 g) and PI (%Energy) increased by one unit, F_2a_-IP (pg/mg creatinine) decreased by 17.34 (*P* < 0.001), and 428.11 (*P* = 0.003), respectively.

**Table 5:** Linear regression model using the GEE method for assessing the association of DII, DTAC, and PI with F_2a_-IP in all participants.

**Variables**	**B**	**SE**	**95% Wald confidence interval** **(lower, upper)**	***P***
DII	28.34	6.45	(15.7, 40.99)	**< 0.001**
DTAC (mmol/100 g)	−17.34	3.76	(−24.72, −9.95)	**< 0.001**
PI (%Energy)	−428.11	142.84	(−708.07, −148.14)	**0.003**

DII, dietary inflammatory index; DTAC, dietary total antioxidant capacity; PI, phytochemical index.

Using the GEE model with identity link function and exchangeable correlation structure (study groups were matched for age and BMI).

Bold p-value (< 0.05) is considered as statistically significant.

## Discussion

In the present study, urinary oxidative biomarkers and their association with the inflammatory and antioxidant potential of diet in male football players and healthy non-athlete controls were investigated. Overall, our findings demonstrated that oxidative stress was significantly lower in football players, and there were also significant relationships between dietary pro-inflammatory and antioxidant indices and oxidative stress.

Long-term and exhaustive exercises are associated with a significant accumulation of oxidative radicals (29). Due to the high energy requirement during exercise, the absorption of oxygen (O_2_) from the blood increases to help muscle contraction. The exercise increases O_2_ consumption in the muscles up to 200 times compared to the resting state (30). It has been concluded that contracting skeletal muscles are an important tissue for ROS production during exercise, and mounting evidence indicates that nicotinamide adenine dinucleotide phosphate (NADPH) is likely to be a major ROS-generating source in contracting skeletal muscle (8, 31). As mentioned previously, the interaction of ROS with the DNA strand and lipids leads to the formation of 8-OHdG and F_2a_-IP, respectively (9, 32).

In the present study, it was indicated that the pro-inflammatory capacity of the diet (assessed by the DII) was significantly lower in athletes compared to non-athletes. Furthermore, the results showed that the DII score could significantly increase 8-OHdG and F_2a_-IP levels, which is consistent with previous similar studies. A study conducted by Moradi et al. reported a positive and negative relationship between DII and malondialdehyde (an OS biomarker) and total antioxidant capacity in healthy people, respectively (33). In addition, it has been demonstrated that a pro-inflammatory diet can induce oxidative processes in the body and that an oxidant-antioxidant imbalance with high levels of nitrogen and ROS can increase DNA damage (34). A study performed by Shahinfar et al. proved that higher DII scores were related to reduced muscle endurance and strength in adults (35). Ramezani et al. also demonstrated that a lower DII is correlated with a higher aerobic capacity (36). Therefore, in football, which relies more on the aerobic pathway (37), a lower DII score may be useful in improving the performance of football players.

The findings showed that the DTAC score was significantly higher in football players compared to non-athletes. Moreover, a negative and significant association was observed among DTAC, 8-OHdG, and F_2a_-IP levels. These findings indicated that DTAC had a significant impact on antioxidant defense against OS caused by exercise. A study conducted on ultra-endurance athletes proved that there was a significant negative correlation between changes in levels of 8-isoprostaglandin F_2a_ and dietary antioxidant intake (FRAP) comparing post-exercise and pre-exercise (19). In addition, Pérez et al. demonstrated an inverse correlation between dietary antioxidants and 8-OHdG (38). Furthermore, a study conducted by Anderson et al. on premenopausal women revealed that there were significant inverse associations between F_2a_-IP and physical activity and dietary antioxidant nutrients (17). In contrast, a study indicated that the composite dietary antioxidant index and dietary antioxidant quality score were not significantly associated with OS (39).

In the current study, the mean dietary antioxidant content calculated with the FRAP assay was 7.79 mmol/day in football players. In the study by Koivisto et al., the FRAP score was considered to be 21.2 mmol/day for an antioxidant-rich diet and 2.8 mmol/day for a low-antioxidant diet in elite endurance athletes (40). Comparing the results of the current study with the findings of the above-mentioned research, it seems the total intake of dietary antioxidants by football players was moderate.

There is evidence suggesting that an antioxidant-rich diet provides several bioactive dietary components called phytochemicals that help to remove ROS and prevent DNA damage by inducing the body's antioxidant defenses (19). It was found that the PI score was higher in football athletes than in non-athletes. Diets containing whole grains, nuts, fresh fruits and vegetables, legumes, and plant foods such as olive oil are rich in phytochemicals, fiber, and antioxidants (41), and the PI score is a simple way to assess phytochemical intake (42). In terms of PI components, there was a significant difference between the two groups of football players and the non-athletes in the intake of fruits, olive oil, tea, and coffee. Furthermore, in the present study, an inverse and significant relationship was observed between the PI score and the 8-OHdG and F_2a_-IP levels. What is more, the findings showed that the PI, compared to DTAC, exerted a greater effect on reducing 8-OHdG and F_2a_-IP. Furthermore, a study showed that a diet rich in phytochemicals can reduce OS (42).

It has been demonstrated that phytochemicals such as flavonoids, carotenoids, and bioflavonoids provide antioxidant support (43). De Carvalho et al. reported that the intake of fruits was inversely related to 8-OHdG, but this relationship was not observed for the consumption of vegetables (44). As mentioned earlier, in this study, there was a significant difference between the two groups in terms of receiving fruits, but no statistically significant difference was observed in terms of vegetable intake. In total, it seems the diet rich in phytochemicals with antioxidant impacts was effective in reducing 8-OHdG and F_2a_-IP levels.

Our results, taken together with those from previous studies, postulated that adherence to a balanced diet rich in natural antioxidants and phytochemicals is the best recommendation regarding exercise and antioxidants in athletes and physically active persons. Regular consumption of various fresh fruits and vegetables, legumes and beans, whole grains, sprouts, and seeds is a safe and effective approach to meeting all antioxidant requirements (45).

The evidence indicates that a diet rich in antioxidants is positively associated with post-exercise blood lactate removal and can increase the running time to exhaustion (19). Therefore, the consumption of a diet rich in antioxidants by football players who are running for a long time can both help to relieve their fatigue faster after exercise and to increase the duration of time they can run without causing fatigue.

In the current study, it was shown that urinary levels of 8-OHdG and F_2a_-IP were significantly lower in football players compared to non-athletes. There is a lot of evidence that supports the hypothesis that physical exercise can increase the production of free radicals and cause OS in the body (46). But as mentioned earlier, regular/moderate exercise can lead to positive consequences such as improving muscle endurance and strength (3, 8). Furthermore, regular exercise can induce adaptive anti-oxidative effects and an improvement of antioxidant capacity that results in a decrease in oxidative markers (22, 47). It is noteworthy that the response of the antioxidant defense to physical effort varies based on exercise type, intensity, duration, and volume (3).

To the best of our knowledge, there is no study investigating the urinary excretion of 8-OHdG in football players, and only one study has evaluated the urinary level of F_2a_-IP in football players, which reported an elevated value of 8-isoprostaglandin F_2a_ soon after the match and returned to baseline within two days' post-match, which is consistent with our results (48). Furthermore, in line with our findings, a meta-analysis study by Tryfidou et al. revealed a trend for decreased DNA damage (8-OHdG) within 5–28 days of post-acute aerobic exercise (11), and also a review study by Nikolaidis et al. reported that regular exercise could lead to decrease in lipid peroxidation (urinary F_2a_-IP) (12). Research shows that supplemental or dietary interventions ameliorate OS (49). The findings of the present study showed the low score of the DII and the high scores of the DTAC and PI in football players. Consequently, lower levels of oxidative biomarkers can be attributed to these dietary indicators and also to adherence to regular football training.

## Strengths and limitations

This study's strengths lie in its novelty, as it is the first study to evaluate both urinary 8-OHdG and F_2a_-IP levels and simultaneously the relationships among DII, DTAC, and PI with 8-OHdG and F_2a_-IP in football players. Moreover, dietary intake was assessed using the valid and reliable FFQ. Moreover, controlling confounding factors such as FM, FFM, and WHR was another strength of the study. Although matching design in participant recruitment was considered and confounding factors such as FM, FFM, and WHR have been adjusted, the impact of better physical fitness on OS levels should also be considered. The FFQ is still the most appropriate tool for collecting subjects' usual intake in the long term, but reporting relied on respondents' memories, which may cause recall bias; nevertheless, we tried to solve these problems by using a trained dietitian to collect data face-to-face. Another limitation is that this study did not assess the level of inflammatory biomarkers due to financial limitations. This suggests that future studies evaluating inflammatory biomarkers should be conducted. Additionally, due to the cross-sectional nature of the study, it is not possible to discuss the mechanisms underlying the results of the present study.

## Conclusion

The present study revealed that urinary excretion rates of 8-OHdG and F_2a_-IP were lower in football athletes compared to non-athletes. In addition, the DII score was lower, and the DTAC and PI scores were higher in football players. The findings showed that diet-related inflammation was correlated with OS levels. As such, dietary factors such as dietary antioxidants and fruit intake had an inverse relationship with OS levels. Furthermore, results indicated that dietary phytochemical intake exerted the greatest effect on decreasing OS biomarkers. Altogether, adherence to an anti-inflammatory and antioxidant-rich diet is likely to be effective for football players due to decreased OS.

## Data availability statement

The original contributions presented in the study are included in the article/supplementary material, further inquiries can be directed to the corresponding author.

## Ethics statement

The studies involving human participants were reviewed and approved by Ethics Committee of Tabriz University of Medical Sciences (Ethical Code: IR.TBZMED.REC.1399.1009). The patients/participants provided their written informed consent to participate in this study.

## Author contributions

MZ conducted research, data analysis, interpretation of the data, and preparation of a draft manuscript. ZS contributed to writing the manuscript. MN contributed to the interpretation of data and the drafting of the manuscript. PS contributed to the data analysis. ME contributed to the study conception and design. BP contributed to the study idea, design, and revision of the manuscript. All the authors read and approved the final manuscript.
